# Characterization, high-resolution mapping and differential expression of three homologous *PAL* genes in *Coffea canephora* Pierre (Rubiaceae)

**DOI:** 10.1007/s00425-012-1613-2

**Published:** 2012-02-21

**Authors:** Maud Lepelley, Venkataramaiah Mahesh, James McCarthy, Michel Rigoreau, Dominique Crouzillat, Nathalie Chabrillange, Alexandre de Kochko, Claudine Campa

**Affiliations:** 1 Nestlé R&D Center, 101 Av. Gustave Eiffel, Notre Dame D’Oé, BP 49716, 37097 Tours, France; 2 IRD, UMR DIADE (IRD/UM2), BP 64501, 34394 Montpellier, France; 3 Avesthagen Limited, International Technology Park, Whitefield Road, Bangalore, 560066 India

**Keywords:** Chlorogenic acids, *Coffea*, Gene expression, Gene structure, Mapping, Phenylalanine ammonia lyase

## Abstract

Phenylalanine ammonia lyase (PAL) is the first entry enzyme of the phenylpropanoid pathway producing phenolics, widespread constituents of plant foods and beverages, including chlorogenic acids, polyphenols found at remarkably high levels in the coffee bean and long recognized as powerful antioxidants. To date, whereas PAL is generally encoded by a small gene family, only one gene has been characterized in *Coffea canephora* (*CcPAL1*), an economically important species of cultivated coffee. In this study, a molecular- and bioinformatic-based search for *CcPAL1* paralogues resulted successfully in identifying two additional genes, *CcPAL2* and *CcPAL3,* presenting similar genomic structures and encoding proteins with close sequences. Genetic mapping helped position each gene in three different coffee linkage groups, *CcPAL2* in particular, located in a coffee genome linkage group (F) which is syntenic to a region of Tomato Chromosome 9 containing a *PAL* gene. These results, combined with a phylogenetic study, strongly suggest that *CcPAL2* may be the ancestral gene of *C. canephora*. A quantitative gene expression analysis was also conducted in coffee tissues, showing that all genes are transcriptionally active, but they present distinct expression levels and patterns. We discovered that *CcPAL2* transcripts appeared predominantly in flower, fruit pericarp and vegetative/lignifying tissues like roots and branches, whereas *CcPAL1* and *CcPAL3* were highly expressed in immature fruit. This is the first comprehensive study dedicated to *PAL* gene family characterization in coffee, allowing us to advance functional studies which are indispensable to learning to decipher what role this family plays in channeling the metabolism of coffee phenylpropanoids.

**Electronic supplementary material:**

The online version of this article (doi:10.1007/s00425-012-1613-2) contains supplementary material, which is available to authorized users.

## Introduction

Over the last few years, an increasing number of studies have focused on the relationship between plant-derived foods containing phenolic compounds (flavonoids/hydroxycinnamic acids and esters) with high antioxidant activity and human health (Lila 2007; Ververidis et al. 2007; Han and Baik 2008; Cuevas-Rodriguez et al. 2010). The growing recognition of the importance of plant antioxidants in human health has thus led to increased research interest in the synthesis and accumulation of these antioxidant compounds in plants (Tamagnone et al. 1998; Hoffmann et al. 2004; Niggeweg et al. 2004; Abdulrazzak et al. 2006; Luo et al. 2008; Vallverdu-Queralt et al. 2011). Both academic and applied interest in this area is further stimulated by the fact that some widely consumed plants are relatively rich in flavonoid/phenolic compounds and by the observation that people who consume higher quantities of these foods appear to have lower risks for certain health problems, such as cardiovascular disease and cancer (Sawa et al. 1999; Bazzano et al. 2002; Clifford 2004; Cos et al. 2005; Go et al. 2005).

The early steps of the phenylpropanoid pathway of plants leading to synthesis of flavonoids and hydroxycinnamic acids and esters, including the large family of chlorogenic acids isomers (CGA), have been described in several plants (Dixon and Paiva 1995; Winkel-Shirley 2002; Clé et al. 2008; Vogt 2010). The first step in the phenylpropanoid pathway is the deamination of phenylalanine to cinnamic acid by l-phenylalanine ammonia lyase (PAL, EC 4.3.1.24). PAL is a tetrameric enzyme whose subunits are encoded by a small multigene family in most species that have been studied (Cramer et al. 1989; Wanner et al. 1995; Fukasawa-Akada et al. 1996; Rasmussen and Dixon 1999). The expression of *PAL* genes are known to be influenced significantly by biotic and abiotic stress (Tovar et al. 2002) and can also be induced during the late plant defense response to pathogens in order to reinforce lignin synthesis in the affected area (Reimers and Leach 1991; Schovankova and Opatova 2011). Four different *PAL* genes have been characterized in *Arabidopsis* and these appear to fall into two different groups (Raes et al. 2003; Cochrane et al. 2004; Huang et al. 2010). As expected for a major branch point between primary and secondary plant metabolic pathways, the expression of the different *Arabidopsis* genes are under complex regulatory control. Three of them (*AtPAL1*, *AtPAL2* and *AtPAL4*) have been shown to be associated with tissue-specific lignin synthesis (Raes et al. 2003; Rohde et al. 2004) and *AtPAL1* and *AtPAL2* appear to be more closely associated with environmental stress-induced flavonoid synthesis (Olsen et al. 2008). *PAL* genes from trees such as poplar have also been studied (Subramaniam et al. 1993; Osakabe et al. 1995; Kao et al. 2002). For example, Kao et al. (2002) reported on the tissue-specific expression of two *PAL* genes from *Populus tremuloides*. *PtPAL1* was found to be more substantially expressed in non-lignifying cells exhibiting accumulation of condensed tannins, and thus more closely connected with their biosynthesis and other phenolics, even if it was also found in developing phloem or xylem. However, *PtPAL2*, expressed in heavily lignified structural cells of shoots, was mostly associated with lignin biosynthesis. Such results strongly suggest that specific *PAL* genes may have distinct and overlapping functions in the phenylpropanoid pathway. Soon after the PAL enzymatic reaction, the phenylpropanoid metabolites generally enter either the flavonoid or lignin synthesis pathways. This step presumably leads to competition for precursors, more especially for coumaroyl-CoA (Mahesh et al. 2006a; Besseau et al. 2007; Clé et al. 2008).

There is currently little published information on the presence of flavonoids in the green or roasted coffee bean and whether these molecules or derivatives thereof contribute to coffee flavor. However, one report suggests that flavonoids are present in roasted coffee (Yen et al. 2005). Whereas other coffee metabolic pathways like those related to caffeine and sucrose have been thoroughly researched (Ky et al. 2001; Privat et al. 2008), there is a lack of information on the phenylpropanoid diversity in coffee. Actually, the main CGA isomers found in the coffee bean are the only compounds synthesized through this pathway whose levels and diversity are well documented in coffee. Based on research literature, these main CGA are represented by 9 out of a total of 30 different isomers identified by Clifford et al. (2006) in the green bean. These main CGA consist of esters formed between one or two *trans*-cinnamic acids (caffeic or ferulic acid) and quinic acid (Clifford 2000), and belong to three classes, each containing three isomers differing in the position of their acyl residues. They include the monocaffeoylquinic acids (CQA; 3CQA, 4CQA and 5CQA), the dicaffeoylquinic acids (diCQA; 3,4diCQA, 3,5diCQA and 4,5diCQA) and the feruloylquinic acids (FQA; 3FQA, 4FQA and 5FQA). Many publications are related to the levels of the main CGA present in green coffee beans, with the corresponding total content found to vary from 7 to 14.4% dry matter in *Coffea canephora* (Ky et al. 1999, 2001; Bertrand et al. 2003; Lepelley et al. 2007; Koshiro et al. 2007) and from 3.4 to 4.8% in *C. arabica* (Ky et al. 2001). These data illustrate the fact that the CGA quantitative diversity is higher in *C. canephora* than in *C. arabica*, whereas no qualitative differences were observed between these two species for this type of compound (Ky et al. 2001).

Due to high amounts of the CGA in coffee beans and thus in the coffee beverage, and to the growing list of health benefits potentially associated with CGA, it is of interest to learn more about how their synthesis/accumulation is controlled on a genetic level. Several authors (Campa et al. 2003; Mahesh et al. 2006a; Lepelley et al. 2007; Koshiro et al. 2007; Joët et al. 2009) have already characterized key genes involved in CGA metabolism in coffee plants, and/or their transcriptional abundance in several tissues. In contrast, there is currently only limited information concerning critical upstream genes like *PAL*, which are essential for regulating the synthesis of CGA precursors. A recent study by Mahesh et al. (2006b) is, to our knowledge, the only report describing a coffee *PAL* gene. In that study, the authors isolated and mapped a *C. canephora PAL* gene (*CcPAL1*) and presented evidence of its role in accumulating certain CGA in *C. canephora*, the caffeoylquinic acids. Here, we describe the isolation of two other *C. canephora* PAL cDNA sequences and their corresponding genomic sequences (*CcPAL2* and *CcPAL3*). Comparison of 98 PAL amino acid sequences from 71 plants has allowed us to build a phylogenetic tree which gives a better view on the relationships existing between the three *C. canephora* proteins and related homologs of other plants, including five proteins from *P. trichocarpa* (Shi et al. 2010a, b), a woody plant whose genome was sequenced and annotated by Tuskan et al. (2006). In addition, we have mapped the three genes on a *C. canephora* consensus map (Lefebvre-Pautigny et al. 2010) and have presented the comparative expression of all three coffee *PAL* genes in different plant organs and tissues at different growth stages to better understand the potential impact of each gene on the synthesis of chlorogenic acids, flavonoids and downstream products such as lignin in coffee.

## Materials and methods

### Plant material

Young leaves of the *C. canephora* BP409 clone were harvested from trees grown in the greenhouse (25°C and 70% relative humidity) at Nestlé R&D facilities in Tours, France, and kept at −80°C before use. *C. canephora* (BP409, 2001) cherries, branches, roots and flowers were harvested from trees cultivated on the experimental farm of ICCRI (Indonesia). The samples were frozen immediately in liquid nitrogen and then sent packed in dry ice to the Nestle R&D Center in Tours, France.

The cherry developmental stages are defined as follows: small green fruit (SG), large green fruit (LG), yellow fruit (Y) and red fruit (R). More details on these stages can be found in Privat et al. (2008). The bean and pericarp tissues of the frozen cherries were rapidly separated for each stage of maturation and then homogenized using a SPEX CertiPrep 6800 Freezer Mill with liquid nitrogen. The powders obtained were then used for RNA extraction.

### RNA preparation and cDNA synthesis for q-RT-PCR experiments

The total RNA was extracted from powdered samples by using a phenol-/chloroform-based method as described previously (Rogers et al. 1999) and treated with DNase to remove DNA contamination as described by Lepelley et al. (2007). Using 1 μg of total RNA and 270 ng poly dT(18) (Eurogentec, Seraing, Belgium) as primers, the method used to make the cDNA was identical to the protocol described in the Superscript II Reverse Transcriptase kit (Invitrogen, Carlsbad, CA, USA) except that the enzyme used was not Superscript II but Superscript III and that the first-strand cDNA-synthesis incubation step was performed for 50 min at 50°C, and not 42°C. The cDNA samples generated were then diluted 100-fold in sterilized water and stored at −20°C for later use in quantitative real-time PCR (q-RT-PCR).

### Quantitative real-time PCR (q-RT-PCR) experiments

Using the cDNA synthesized as described above, quantitative PCR with TaqMan probes and primers was carried out as described previously by Simkin et al. (2006), except that the cDNA dilutions and the Taqman primers/probes were different. The TaqMan^®^ probe-based chemistry uses a fluorogenic probe to enable the detection of a specific PCR product as it accumulates during PCR. The primers and TaqMan probes used were designed with the Primer Express software (Applied Biosystems, Foster City, CA, USA) and are listed in Supplementary Table S1. Each primers/probe set was designed to be specific to the target gene to avoid the amplification of other related genes. Moreover, the last five bases on the 3′ end of the primers were designed to contain no more than two C and/or G bases, which is another factor that prevents the possibility of non-specific PCR product formation. The amplification efficiency of the primer/probe sets was tested on various dilutions of the corresponding plasmids and were all found to be near 100%. Quantification was conducted using the GeneAmp 7500 Sequence Detection System (Applied Biosystems) and repeated three times for each reaction. Transcript levels were normalized to the levels of the constitutively expressed ribosomal *rpl39* gene.

### Primer design and PCR amplification for cDNA isolation

Primer design was performed based on *C. canephora* cDNA sequences available in the EST databases developed in the IRD laboratory (Poncet et al. 2006). For *CcPAL2*, a reverse primer *PALBR* (5′-GGACAAGATCACCTGATGCAGT-3′) was designed to amplify the 5′ end. PCRs were carried out using GoTaq DNA polymerase (Promega, Madison, WI, USA) following the manufacturer’s recommendations. Both cDNA libraries (derived from fruit or from leaf) were used as matrices for a PCR reaction using a combination of the *PALBR* gene-specific primer and the universal T3 vector-specific primer. PCR cycles were as follows: 5 min at 94°C, followed by 30 cycles of 50 s at 94°C, 50 s at 52°C, and 50 s at 72°C, and then 10 min at 72°C.

For *CcPAL3*, a reverse primer *PALCR2* (5′-GCGACTTGGTGAACTGTGCTCTTG-3′) was designed to amplify the 5′ end. As for *CcPAL2* isolation, PCR reactions were run as described above on both cDNA libraries using the combination of the reverse primer *PALCR2* and a vector-specific T3 primer, under the following conditions: 5 min at 94°C, followed by 30 cycles of 50 s at 94°C, 50 s at 52°C, and 50 s at 72°C, and then 10 min at 72°C.

### Primer design and PCR amplification for gene structure

To define the position, length and sequence of *PAL* introns, *C. canephora* genomic DNA (~100 ng) was extracted from leaves of trees maintained in tropical greenhouses at IRD Montpellier using the DNeasy Plant Mini-Kit (Qiagen, Hilden, Germany). Primers were designed in the first exon for the forward primer and in the second exon for the reverse one, closer to the intron, assuming that all the *C. canephora* *PAL* genes have their intron at the same position, the reference being *CcPAL1* (Mahesh et al. 2006b). For *CcPAL2*, the primers *PALBF1* (5′-GGTGCCCTTCAGAAGGAGCTTATT-3′) and *PALBR* (5′-GGACAAGATCACCTGATGCAGT-3′) were used in presence of GoTaq (Promega). PCR conditions were: 5 min at 94°C, followed by 29 cycles of 40 s at 94°C, 50 s at 52°C and 1 min at 72°C, and then 10 min at 72°C. The forward primers *PAL1*F (5′-GTTACGGTATCACCACCGGCTTTG) and *PALD*F1 (5′-CATTCCAATCGCTTCTGCGTCC-3′) and the reverse primers *PAL*R2 (5′-TAGAAGCCCTTGTTGCCGAGTGAG) and *PALDR1* (5′-CGTGTGACCTGACTCTGTTCCAT-3′) were used for *CcPAL1* and *CcPAL3*, respectively, and the PCR conditions were: 5 min at 94°C, followed by 29 cycles of 40 s at 94°C, 50 s at 52°C and 1 min at 72°C, and then 10 min at 72°C.

### Subcloning and sequencing

The PCR products were checked on 1% (w/v) agarose gels after ethidium bromide staining and directly purified (QIAquick PCR Purification Kit, Qiagen) for sequencing (MWG-Biotech/Eurofins; Nantes, France) or cloned into a TOPO-TA vector (Invitrogen) and transformed into One Shot© TOP10 competent cells. Plasmid DNA was isolated from overnight cultures using the QIAprep Spin Miniprep protocol (Qiagen) and sequenced.

### Phylogenetic analysis

The amino acid sequences of PALs from fungi, gymnosperms and angiosperms were searched in public databases available at NCBI (http://www.ncbi.nlm.nih.gov). Only full amino acid sequences were used (length of about 700 AA), with a preference for species in which more than one gene was described. Phylogenetic analysis was conducted using Geneious software (5.5.5 version; Biomatters Ltd, Auckland, New Zealand) and the neighbor-joining method was used to build the phylogenetic tree (Drummond et al. 2011). Bootstrap analysis was performed using 1,000 replicates. The accession numbers and the sequences names, as used in Fig. 2, are also reported in Supplementary Table S2 which also contains the full name of the species from which the sequences were isolated.

### Coffee genetic mapping

Using high-resolution melting (HRM) technology, *CcPAL1*, *CcPAL2* and *CcPAL3* genes were mapped on the *C. canephora* COSII (Conserved Ortholog Set) genetic map (BP409 × Q121) published by Lefebvre-Pautigny et al. (2010). Primer pairs were designed for each *PAL* sequence to obtain an amplified DNA fragment length between 200 and 350 bp: PAL1-HRM-FP (GGGAGAGTTGGGGACCAAT) and PAL1-HRM-RP (TTCAACATTTATGGCAACGAAC) as the forward and reverse primers for amplifying a 321-bp DNA fragment specific to *CcPAL1* gene; PAL2-HRM-FP (ACTGCTGACTGGAGAGAAAG) and PAL2-HRM-RP (TGGGTGTTACAGACATATCA) as the forward and reverse primers for amplifying a 228-bp DNA fragment specific to *CcPAL2;* and finally PAL3-HRM-FP (AGAGCTGAAGACCCTTTTGC) and PAL3-HRM-RP (CTCTTCTGTCGGCCTTCAC) as the forward and reverse primers for amplifying a 344-bp DNA fragment specific to *CcPAL3.*

As described for the HRM assays referred to by Lefebvre-Pautigny et al. (2010), the reagent components for one genotyping assay were added to the 20-μl final reaction volume as follows: 2 μl of genomic DNA (20 ng), 10 μl of Master Mix (2× containing fluorescent dye), 1 μl of primer mix (final concentration of 0.2 μM for each primer), 2.4 μl of MgCl_2_ and 3.6 μl of sterilized water. Then, PCR reactions were run using the Lightcycler^®^ 480 Thermocycler (Roche). The PCR conditions were as follows: 10 min at 95°C, followed by 45 cycles of 10 s at 95°C, 15 s at 60°C and 25 s at 72°C then followed by a last denaturing step of 1 min at 95°C, 1 min at 40°C (renaturation), and finally ending with a ramp rate of 0.02°C/s from 60 to 95°C (melting). During this last step, fluorescence is read 25 times per degree. Analyses of DNA melting curves and allele segregations were performed using “Gene Scanning” software (Roche).

### Bioinformatic analysis

Determination of percentage identities shared between CcPAL1, CcPAL2 and CcPAL3 protein sequences and their closely related sequences in several plant species.

Using NCBI as the worksite, each of the three coffee PAL protein sequences discussed here was used as a query of a BLASTP search against NCBI non-redundant databases, using the default alignment parameters. Several of the best hits found were selected and reported in Table 1. The percentage of identity (PIs) shared between the protein sequences of these hits along with the respective coffee PAL protein sequences, the alignment scores and the *e* values resulting from these alignments are also shown in Table 1.

**Table 1:** Amino acid sequence identities shared between CcPAL1 (accession number #AAN32866), CcPAL2 (accession number #AEO94540) and CcPAL3 (accession number #AEO94541) protein sequences and some of their best hits found, plus *Arabidopsis thaliana* PAL hits—from a BLASTP search using the coffee sequences as queries against the non-redundant database of NCBI

Species	Sequence	Sequence accession number	Identity (%)	*e* value	Score (bits)
*Coffea canephora Cc*PAL1 hits (CcPAL1 accession number #AAN32866)					
*Populus trichocarpa*	PtrPAL5	ACC63889	87	0.0	1264
*Manihot esculenta*	MePAL1	AAK62030	86	0.0	1258
*Vitis vinifera*	VvPAL	ABM67591	85	0.0	1269
*Camellia sinensis*	CsPAL	BAA05643	84	0.0	1251
*Citrus limon*	ClPAL	AAB67733	84	0.0	1254
*Manihot esculenta*	MePAL2	AAK60275	84	0.0	1248
*Coffea canephora*	CcPAL2	AEO94540	83	0.0	1235
*Coffea canephora*	CcPAL3	AEO94541	83	0.0	1200
*Arabidopsis thaliana*	AtPAL1	AEC09341	82	0.0	1208
*Arabidopsis thaliana*	AtPAL4	AEE74893	82	0.0	1180
*Arabidopsis thaliana*	AtPAL2	AEE79055	81	0.0	1213
*Arabidopsis thaliana*	AtPAL3	AED90714	75	0.0	1050
*Coffea canephora Cc*PAL2 hits (CcPAL2 accession number #AEO94540)					
*Catharanthus roseus*	CrPAL	BAA95629	88	0.0	1312
*Ipomoea nil*	InPAL	AAG49585	88	0.0	1302
*Nicotiana tabacum*	NtPAL1	BAA22948	88	0.0	1300
*Nicotiana tabacum*	NtPAL2	BAA22963	87	0.0	1295
*Capsicum annuum*	CaPAL	ACF17667	88	0.0	1291
*Daucus carota*	DcPAL	BAC56977	86	0.0	1291
*Arabidopsis thaliana*	AtPAL1	AEC09341	83	0.0	1224
*Arabidopsis thaliana*	AtPAL2	AEE79055	82	0.0	1210
*Coffea canephora*	CcPAL3	AEO94541	82	0.0	1197
*Arabidopsis thaliana*	AtPAL4	AEE74893	82	0.0	1187
*Arabidopsis thaliana*	AtPAL3	AED90714	73	0.0	1041
*Coffea canephora Cc*PAL3 hits (CcPAL3 accession number #AEO94541)					
*Camellia oleifera*	CoPAL	ACT21093	85	0.0	1255
*Populus trichocarpa*	PtrPAL2	ACC63890	85	0.0	1232
*Jatropha curcas*	JcPAL	ABI33979	84	0.0	1248
*Ricinus communis*	RcPAL	EEF42935	84	0.0	1243
*Vitis vinifera*	VvPAL	ABM67581	84	0.0	1234
*Manihot esculenta*	MePAL1	AAK62030	84	0.0	1224
*Manihot esculenta*	MePAL2	AAK60275	83	0.0	1231
*Catharanthus roseus*	CrPAL	BAA95629	83	0.0	1226
*Pyrus communis*	PcPAL	ABB70117	82	0.0	1232
*Arabidopsis thaliana*	AtPAL2	AEE79055	81	0.0	1188
*Arabidopsis thaliana*	AtPAL4	AEE74893	81	0.0	1178
*Arabidopsis thaliana*	AtPAL1	AEC09341	79	0.0	1191
*Arabidopsis thaliana*	AtPAL3	AED90714	74	0.0	1042

### Genbank accession numbers

Sequence data from this article have been deposited into GenBank under the following accession numbers: *C. canephora* CcPAL1 cDNA (AF460203), *C. canephora* CcPAL2 cDNA (JF805760) and *C. canephora* CcPAL3 cDNA (JF805761); *C. canephora* CcPAL1 protein (AAN32866), *C. canephora* CcPAL2 protein (AEO94540) and *C. canephora* CcPAL3 protein (AEO94541); *C. canephora* CcPAL1 genomic DNA (JN420343), *C. canephora* CcPAL2 genomic DNA (JN420344) and *C. canephora* CcPAL3 genomic DNA (JN420345). Note that, as additional information, *C. arabica* CaPAL1 and CaPAL2 cDNA sequences were also deposited under accession numbers JF838179 and JF838180, respectively.

## Results

### Isolation and characterization of *PAL* homologous genes in *C. canephora*

Screening both partial *C. canephora* EST databases developed at IRD (Poncet et al. 2006) allowed us to identify two partial cDNA sequences (*CcPALB* and *CcPALD*) encoding putative phenylalanine ammonia lyase (PAL). These sequences differed from that of *CcPAL1*, but were truncated at the 5′ end. Reverse PCR performed on both leaf and fruit cDNA libraries helped to amplify several fragments. The longest cDNA sequences were found to represent two new full-length cDNA clones which were called *CcPAL2* and *CcPAL3*.

Analysis of the open reading frames of the new cDNAs showed that both sequences were slightly shorter than that of *CcPAL1*, with *CcPAL2* and *CcPAL3* being 2,136-bp and 2,142-bp long, respectively (vs. 2,154 bp for *CcPAL1*). They encoded polypeptides of 711 and 713 amino acids, respectively (717 AA for CcPAL1). The comparison of the three CcPAL amino acid sequences is presented in Supplementary Fig. S1. The predicted molecular mass and theoretical p*I* of the two new coffee PALs proteins (77.2 kDa and 6.14; 77.7 kDa and 6.54, respectively) are consistent with the size determined for PAL polypeptides from other plants which vary from 72 to 83 kDa (reviewed in Hahlbrock and Grisebach 1979). The predicted AA sequences showed the expected conserved patterns found in all other PAL sequences, in particular those at the PAL active site: G-[STG]-[LIVM]-[STG]-[AC]-S-G-[DH]-L-x-P-L-[SA]-x(2)-[SAV] (Schuster and Rétey 1994). By using parsley PAL as a model, Schuster and Rétey (1994) predicted that the active site was associated with a serine residue that had been converted to dehydroalanine. This serine residue is conserved among the species and is located at the following positions from the first methionine: 204 in CcPAL1, 198 in CcPAL2 and 201 in CcPAL3. The deduced amino acid sequences of CcPAL1 and CcPAL2 shared 83% identity. There is 83% identity between PAL1 and PAL3 and 82% identity between PAL2 and PAL3 (Table 1). As noted in other PAL protein sequences, the maximum divergence was found at the N-terminal extremities.

**Fig. 1:**
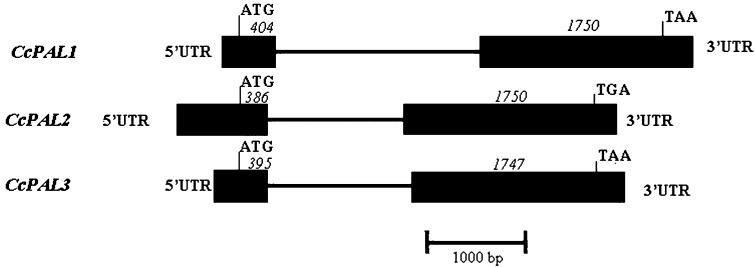
Representation of the structure of the *PAL* gene family in *Coffea canephora.* Exons and untranslated regions (UTRs) are shown as *boxes* and introns are indicated as *lines*

Analyzing the corresponding genomic clones revealed that, as expected and similarly to *CcPAL1*, both *CcPAL2* and *CcPAL3* contained only one intron. This intron was positioned at 404, 386 and 395 bases from the ATG for *CcPAL1*, *CcPAL2* and *CcPAL3*, respectively. Its length varied from 1,263 bp for *CcPAL2* to 1,338 bp for *CcPAL3* and 1,903 bp for *CcPAL1* (Fig. 1). Curiously, *CcPAL1* had a phase 2 intron, the rarest in eukaryotes (Fedorov et al. 1992), and both *CcPAL2* and *CcPAL3* have a phase 1 intron. In genomic *PAL* sequences of other plants, a single intron was also observed, its length varying from 90 bp in *Pisum sativum* (Yamada et al. 1992) to 1,900 bp in *Nicotiana tabacum.* In angiosperms, similarly to the three genes from *C. canephora* already observed, this intron is generally positioned between the second and third bases of a conserved Arg codon. The cDNA sequences showed that the exon–intron junctions in the *CcPAL* genes maintained the “GT-AG” rule for donor/acceptor sites (Breathnach and Chambon 1981). The first exon varied in length, encoding 135 AA, 129 AA or 132 AA in *CcPAL1*, *CcPAL2* and *CcPAL3,* respectively. The length of Exon 2 is conserved in *CcPAL1* and *CcPAL2* paralogs (1,750 bp) and that of *CcPAL3* was remarkably close (1,747 bp).

**Fig. 2:**
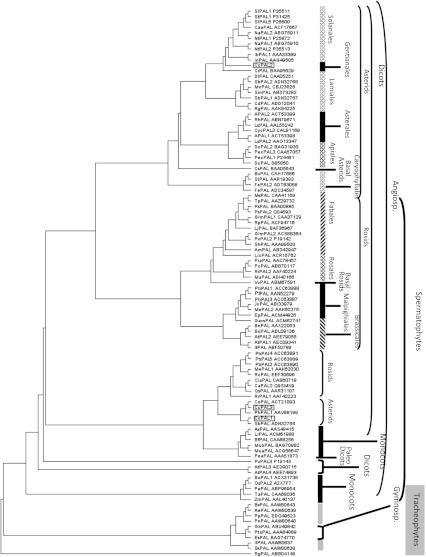
Phylogenetic relationships between the *Coffea canephora* PAL proteins and PAL proteins from spermatophytes (dicots, monocots and gymnosperms) and tracheophytes using *Rhodotorula glutinis* PAL protein sequence as an outgroup (see Supplementary Table S2)

A high level of identity (over 80%) was observed when comparing the deduced amino acid sequence of the three coffee genes with PAL sequences from other plants (Table 1). The highest identity was obtained when comparing *C. canephora* CcPAL2 with *Catharanthus roseus, Ipomea nil, N. tabacum* and *Capsicum annuum* PAL sequences (88% identity). The CcPAL3 protein sequence shared less identity (83%) with this *C. roseus* PAL amino acid sequence (#BAA95629). A rooted phylogenetic tree for PAL protein families from several different species was constructed using the three PALs from *C. canephora* and 97 PALs from other plants. A PAL amino acid sequence from the fungus (*Rhodothorula glutinis*) was used as the root (Fig. 2). CcPAL1 and CcPAL3 sequences were found distant, however, from CcPAL2, which was more closely related to the *C.* *roseus* PAL sequence discussed above. This plant, like the coffee tree, is a member of the Gentianales, an order of flowering plants which belongs to the Asterid clade (APG II classification). In fact, 34 sequences out of the 39 analyzed belonging to the Asterids are branched together. The same observation can be made for 29 PAL sequences among the 41 analyzed for the Rosids. Only 17 sequences among the 70 from Eudicots and a Basal dicot (*Persea americana*) are grouped with some monocots. Most of them actually appeared to be PAL sequences deduced from genes that possess homologs in the Rosid or Asterid branches of the tree. For example, *P. trichocarpa*, *Phaseolus vulgaris*, *Manihot esculenta*, *Arabidopsis thaliana* (Rosids) or *Scutellaria baicalensis* (Asterids) had PAL sequences close to *C. canephora’s* PAL1 and 3, but these species presented other PAL sequences that closely matched the order of the species that they belong to: Fabales, Malpighiales and Brassicales, respectively, for the Rosids; Lamiales for the Asterids. The phylogenetic tree (Fig. 2) also shows that the five PAL proteins from *P. trichocarpa* (PtrPALs) are clustered into two different phylogenetic groups, which is consistent with the results obtained by Shi et al. (2010a, b), who also classified them into two phylogenetic groups, each subset having a tissue-specific expression shared between the members belonging to it: subset A (PtrPAL2, PtrPAL4 and PtrPAL5) and subset B (PtrPAL1 and PtrPAL3). It is interesting that from the tree, the two PtrPALs from subset B are shown to be very close to other Malpighiales protein sequences and are phylogenetically closer to CcPAL2 than to the other two CcPALs and three PtrPALs proteins, whereas the three PtrPALs from subset A are phylogenetically closer to CcPAL1 and CcPAL3. Protein sequences such as CcPAL2, PtrPAL1 and PtrPAL3 whose phylogenetic relationships are comparable to those given by the phylogenetic classification APGII might therefore correspond to sequences encoded by orthologous genes derived from the same ancestor. The other PAL protein sequences issued from these species (CcPAL1 and CcPAL3 for coffee and PtrPAL2, PtrPAL4 and PtrPAL5 for poplar) may result from gene duplication events leading to paralogous genes, which may have occurred after the divergence from the common ancestor. By observing similar grouping when comparing PtrPAL protein sequences and *PtrPAL* specific gene expression (Shi et al. 2010a, b), we may deduce that the differences observed in the promoter region and in the coding sequence from a specific subset resulted from the same duplication event. However, the fact that *P. trichocarpa* proteins from the subset B grouped with PAL proteins from plants that are not woody angiosperms, such as Euphorbiaceae, leads us to speculate that the xylem specificity of this subset would probably not be related to the protein sequences, but rather to the promoter sequences.

### Tissue-specific expression of *PAL* genes in *C. canephora*

**Fig. 3:**
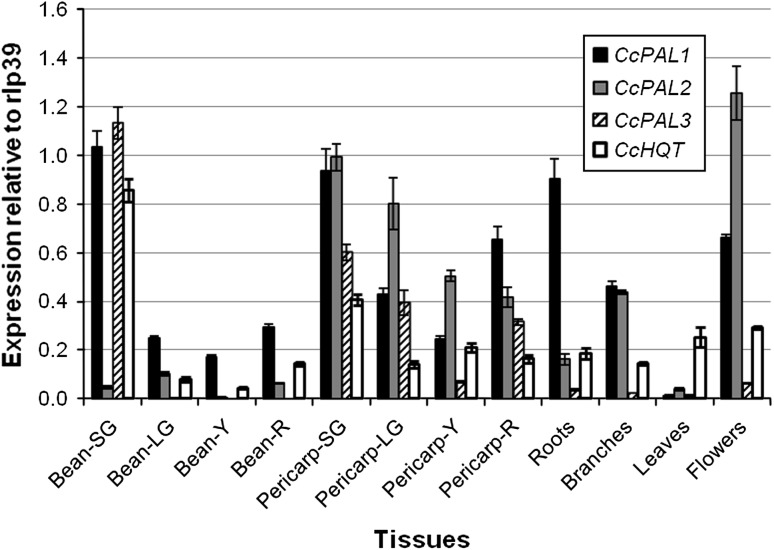
Relative expression of *CcPAL1*, *CcPAL2*, *CcPAL3*, three *PAL* family members and of *HQT* in different tissues and organs of a *Coffea canephora* variety (Robusta BP409). The gene expression relates to the constitutively expressed gene *RPL39*. *Bean-SG* small green-stage bean, *Bean-LG* large green-stage bean, *Bean-Y* yellow-stage bean, *bean-R* red-stage bean, *Pericarp-SG* small green-stage pericarp, *Pericarp-LG* large green-stage pericarp, *Pericarp-Y* yellow-stage pericarp, *Pericarp-R* red-stage pericarp

The transcript levels of *CcPAL1*, *CcPAL2* and *CcPAL3* were measured in several tissues of the Robusta variety BP409 using q-RT-PCR. The three *PAL* genes were expressed in nearly all the tissues examined, but the transcript levels showed significant variations depending on the *PAL* gene and/or the tissue and organ (Fig. 3). In the bean, the relative expression data for the three *PAL* genes highlighted that the *CcPAL1* and *CcPAL3* genes were highly and similarly expressed at the more immature stage with RQ_PAL1(Bean-SG)_ = 1.03 and RQ_PAL3(Bean-SG)_ = 1.13, whereas expression of the *CcPAL2* gene was nearly 20 times lower at the same stage with RQ_PAL2(Bean-SG)_ = 0.045). As bean development progresses, *CcPAL3* expression falls to nearly undetectable levels. This observation suggests that the corresponding protein is needed at high levels for bean metabolism at the early stage (small green) and also possibly at the next stage (LG, large green fruit). It will be interesting in the future to determine if this protein is present after this point of development using Western blotting. In contrast to *CcPAL3, CcPAL1* and *CcPAL2* transcripts are found at reasonable levels in the later stages of bean development, with *CcPAL1* and *CcPAL2* showing RQ = 0.25 and RQ = 0.10, respectively, in the LG stage.

All three PAL genes in the pericarp are expressed at their highest level at the more immature stage (RQ_PAL1(Pericarp-SG)_ = 0.94, RQ_PAL2(Pericarp-SG)_ = 0.99 and RQ_PAL3(Pericarp-SG)_ = 0.60). Then, the transcript levels decrease progressively through the next two maturation stages (LG and Y) and stabilize at the most mature (R) stage for *CcPAL2*, whereas *CcPAL1* and *CcPAL3* expressions increase slightly. This latter observation suggests that *CcPAL1* and *CcPAL3* could play a significant role in the final red fruit/pericarp coloration at the mature stage, a color which is probably due to the synthesis of certain flavonoids/anthocyanins.

Regarding expression in vegetative tissues, relatively low transcript levels were found for all three *CcPALs* in leaves, but significant levels of *CcPAL1* and *CcPAL2* transcripts were found in the roots, branches and flowers, with the higher expression found in the flowers being attributed to *CcPAL2* (RQ_PAL2(Flowers)_ = 1.26), the highest expression found in the roots being attributed to *CcPAL1* (RQ_PAL1(Roots)_ = 0.9) and similar transcript levels detected for *CcPAL1* and *CcPAL2* in the branch (RQ ≈ 0.45). Thus, *CcPAL1* and *CcPAL2* are likely to make important contributions to vegetative tissue phenylpropanoid metabolism, whereas *CcPAL3* appears to have a much smaller role in the bulk of these tissues.

### *CcPAL* map location on a *C. canephora* map

**Fig. 4:**
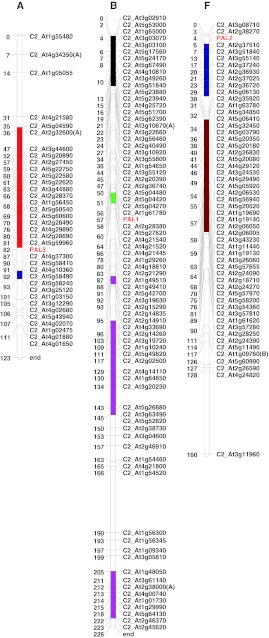
Genetic mapping of *CcPAL1*, *CcPAL2* and *CcPAL3* on the coffee COSII “synteny” map. The letters A, B and F represent coffee linkage groups A, B and F. Shared regions of synteny between coffee and tomato are shown as *colored blocks*, and were deduced by COSII loci mapping in both species (Lefebvre-Pautigny et al. 2010). Each region sharing synteny with tomato is marked by a different color (modified from Wu et al. 2009). Shared regions of synteny with tomato chromosomes are indicated by: *red* for chromosome 11, *blue* for chromosome 9, *black* for chromosome 3, *green* for chromosome 2, *pink* for chromosome 1 and *brown* for chromosome 7

Using HRM technology, the three *PAL* genes were mapped on the *C. canephora* COSII genetic map published by Lefebvre-Pautigny et al. (2010). This genetic map includes 396 COSII markers allowing for syntenic studies among plant species. As shown in Fig. 4, each *CcPAL* gene was mapped in a different linkage group, confirming that at least three distinct *CcPAL* genes are present in the *C. canephora* genome. Cc*PAL1,* *CcPAL2* and *CcPAL3* are, respectively, located on coffee linkage groups B, F and A.

As in another result shown in Fig. 4 and based on Lefebvre-Pautigny et al. (2010), the *CcPAL2* gene is the only one of the three coffee *PAL* genes to be located in a coffee chromosome region that was found to be syntenic to the tomato genome. More precisely, the coffee syntenic genome area carrying *CcPAL2* (3–23 cM) on linkage group F is found to be syntenic with a fragment of the tomato chromosome 9 (0.5–24 cM). In addition, and thanks to tomato maps available through the SGN Web site hosted at the Boyce Thompson Institute (New York, USA) (http://solgenomics.net/), it was discovered that this tomato chromosome region contains an RFLP-type tomato marker (CT225) that also encodes a PAL protein.

## Discussion

Searching for *CcPAL1* paralogs in *C. canephora* successfully led to the isolation of two new coffee *PAL* genes, *CcPAL2* and *CcPAL3*, which differed by the encoded proteins and their location on the *C. canephora* genetic map (Lefebvre-Pautigny et al. 2010). In addition, a complementary bioinformatic analysis aimed at establishing the number of *PAL* genes expressed in the coffee plant was conducted to screen the publicly available *C. canephora* EST databases (Lin et al. 2005; http://solgenomics.net/). This study detected the same three genes, but found no other potential *PAL* gene. The resulting mapping data obtained for the three genes will be integrated into a study aiming to identify the quantitative trait loci (QTL) affecting agronomically important traits in coffee beans. If these genes are found to co-localize with the identified QTL, they will likely be used as candidates for improving coffee plants through marker-assisted selection (MAS) (Srinivas et al. 2009; Mohan et al. 2010). The presence of these three expressed *PAL* paralogs in *C. canephora* is consistent with results already obtained for other plant species. Indeed, *PAL* genes generally belong to a multigene family whose number of genes varies depending on the plant considered. For example, *A. thaliana* contains four *PAL* genes: *AtPAL1* → *AtPAL4* (Raes et al. 2003). But some plant species, such as the potato or particularly the tomato, contain several decades of *PAL* genes among which many are inactive (Joos and Hahlbrock 1992; Chang et al. 2008). It would thus be interesting to determine whether such transcriptionally inactive *PAL* genes could also exist in coffee, which would not be surprising, given the fact that coffee and tomato share common gene repertories (Lin et al. 2005). The forthcoming genome sequence of *C. canephora* will certainly help answer that question. Nevertheless, even when degenerated primers were used to amplify PAL sequences, only the same three transcriptionally active *PAL* genes described here and in a previous study (Mahesh et al. 2006b) were amplified.

A previous study suggested that *CcPAL1* may be involved in the differential accumulation of the main groups of CGA found in green coffee beans, the caffeoylquinic acids (Mahesh et al. 2006b). However, the quantitative gene expression results obtained in the present work using Taqman q-RT-PCR technology, while consistent with the involvement of *CcPAL1* in CGA accumulation in coffee beans, also strongly suggest that *CcPAL2* and *CcPAL3* are likewise involved in this process. Indeed, *PAL* gene expression analysis in various coffee tissues showed that two or three coffee *PAL* genes are often expressed in a single tissue or organ, but not necessarily at the same stage of development. In the bean, for example, *CcPAL1* and *CcPAL3* were found to be highly expressed at the immature stage, while the *CcPAL2* expression was extremely low. Yet, later in maturation (from LG stage), *CcPAL1* and *CcPAL3* transcript levels had fallen significantly and the *CcPAL2* transcript levels had risen slightly. The high expression of *CcPAL1* and *CcPAL3* at the small green stage correlates with the high co-expression of *HQT* (*hydroxycinnamoyl*-*CoA quinate hydroxycinnamoyl transferase*), a gene also involved in CGA biosynthesis (Niggeweg et al. 2004). This observation suggests that the concomitant high expression of these genes leads to a high production of CGA during the SG stage (Lepelley et al. 2007). In addition, the *CcPALs* and *CcHQT* transcript levels tend to fall as the quantity of CGA drops in the later stages of bean development, suggesting that the genes are co-regulated.

The fact that all three *CcPAL* transcripts were detected in many tissues or organs, but at quite varying levels, suggests that the corresponding enzymes probably play particular roles in different parts of the plant while simultaneously playing smaller overlapping housekeeping roles. While *CcPAL1* and *CcPAL3* appear to be more strongly linked with a high accumulation of CGA in coffee bean, it appears that *CcPAL2* may contribute more significantly to flavonoid accumulation. Evidence for this proposal comes from the substantial level of expression of *CcPAL2* in the flower, an organ known to have relatively important rate of flavonoid synthesis. The fact that *CcPAL1* transcription level was also high suggests that this gene could also contribute to the flavonoid precursor pool in this organ. Finally, it is also important to note that the three coffee *PAL* genes are expressed when the pericarp of the coffee cherry is red, suggesting that they may participate in fruit coloration. Searching for their potential co-expression with others genes, either from the general phenylpropanoid pathway (i.e., *C4H* and *4CL*), or from the specific flavonoid (i.e., *CHS* and *CHI*) or lignin (i.e., *COMT* and *CAD*) branches, could help to determine more precisely whether a coffee *PAL* could be more specifically involved in one of the two major phenylpropanoid branches. Such an approach was used by Gachon et al. (2005), who showed that in *Arabidopsis* the paralogs of the phenylpropanoid pathway displayed a clear differential co-expression according to the culture conditions applied to the plant and its response. In the same manner, Mahroug et al. (2006) linked the specific co-expression observed for three genes (*PAL*, *C4H* and *CHS*) with the high flavonoid content found in the upper epidermis of *C. roseus*.

The three coffee PAL-deduced protein sequences were compared to 98 PAL protein sequences from other species. The phylogenetic tree helped to clarify that CcPAL2 was the only one of the three proteins whose sequence was branched with other PAL sequences from the Asterids, the clade to which *C. canephora* belongs. *CcPAL2* has been located within a coffee linkage group region that is syntenic to tomato (observation based on the results obtained by Lefebvre-Pautigny et al. 2010). This observation strongly suggests that *CcPAL2* and the corresponding tomato *PAL* gene both derive from a common *PAL* ancestor as do, most probably, the other Asterid genes whose protein-derived sequence branched together on the phylogenetic tree (Fig. 2). *CcPAL1* and *CcPAL3* could be specific *Coffea* paralogs produced from an ancient duplication of *CcPAL2,* resulting in one of the two genes being followed by a more recent duplication of the duplicated paralog, since *CcPAL1* and *CcPAL3* seem to branch very closely on the phylogenetic tree. Both *CcPAL2* and *CcPAL3* have a phase 1 intron. *CcPAL2* being the ancestral form, it might be assumed that *CcPAL3* results from the first duplication. As *CcPAL1* carries a phase 2 intron, it may then be alleged to be the most recent paralog, resulting from a duplication of *CcPAL3*. Interestingly, CcPAL1 and CcPAL3 proteins were found grouped with PtrPAL2, PtrPAL4 and PtrPAL5, three *P. trichocarpa* proteins encoded by genes more specifically expressed in xylem and carrying, in their promoters, five core motifs similar to elements which are known to regulate phenylpropanoid gene expression (Shi et al. 2010a, b). This result suggests that *CcPAL1* and *CcPAL3* may be co-expressed with genes of the phenylpropanoid pathway that lead to monolignol biosynthesis. As both paralogs seemed to be the product of duplications, lignification in woody plants could be considered as a derived function from an ancestral one from the phenylpropanoid pathway, such as flavonoid biosynthesis, the first plant protection against UV light. This observation highlights that it would be informative to isolate the 5′ untranslated transcribed region (UTR) and the promoter region sequences of the three *CcPAL* paralogs to acquire meaningful additional data on their specific functions and their transcriptional control, often exercised by *R2R3*-*MYB* transcription factors in monolignol and flavonoid synthesis (Stracke et al. 2007; Bomal et al. 2008; Luo et al. 2008).

The present work, dedicated to the family of coffee *PAL* genes, has successfully led to identifying, characterizing and mapping three genes. Their differential expression, and particularly the association established between the high expression observed for *PAL1*, *PAL3* and *HQT* genes (Fig. 3) and the high CGA accumulation in the immature coffee bean, is a preliminary step toward characterizing their functions. In further research, it would be interesting to assess if all three coffee PAL proteins are biochemically active in vitro, by producing them in *Escherichia coli*, and then testing their ability to catalyze the deamination of l-phenylalanine to form *trans*-cinnamic acid (Reichert et al. 2009). As a key step, studying coffee *PAL* expression profiles and segregation in different *C. canephora* varieties and in their offspring after crossing, and quantifying the related phenylpropanoid levels for association studies, will be particularly useful for advancing coffee breeding programs. The use of such genetic markers encoding proteins associated with flavonoids and CGA accumulation could carry substantial interest for the selection of *C. canephora* varieties with improved traits, e.g., varieties rich in antioxidant compounds beneficial to human health or with improved organoleptic quality.

## Electronic supplementary material

Below is the link to the electronic supplementary material.

Supplementary material 1 (PDF 243 kb)

Supplementary material 2 (PDF 269 kb)

Supplementary material 3 (PDF 300 kb)

## Abbreviations

CGA: Chlorogenic acids
COS: Conserved ortholog set
EST: Expressed sequence tag
HQT: Hydroxycinnamoyl-CoA quinate hydroxycinnamoyl transferase
HRM: High-resolution melting
MAS: Marker-assisted selection
PAL: Phenylalanine ammonia lyase
QTL: Quantitative trait loci
q-RT-PCR: Quantitative real-time polymerase chain reaction
