# Supplementary material for: Characterization, high-resolution mapping and differential expression of three homologous PAL genes in Coffea canephora Pierre (Rubiaceae)
Source: Planta. 2012 Feb 21;236(1):313–26. doi: 10.1007/s00425-012-1613-2 (PMC3382651; doi:10.1007/s00425-012-1613-2)
Supplement: Supplementary file 1 — Supplementary material 1 (PDF 243 kb) [file 425_2012_1613_MOESM1_ESM.pdf]

Characterization, high-resolution mapping and differential expression of three homologous *PAL* genes in *Coffea canephora* Pierre (Rubiaceae).

Maud Lepelley[1,*], Venkataramaiah Mahesh[2,3], James McCarthy[1], Michel Rigoreau[1], Dominique Crouzillat[1], Nathalie Chabrillange[2], Alexandre de Kochko[2], Claudine Campa[2]

[1] Nestlé R&D Center, 101 Av. Gustave Eiffel, Notre Dame D'Oé, BP 49716, 37097 Tours, France

[2] IRD, UMR DIADE (IRD/UM2), BP 64501, 34394 Montpellier, France

[3] Avesthagen Limited, International Technology Park, Bangalore 560066, India

* Corresponding author E-mail Address: maud.lepelley@rdto.nestle.com

```
PAL1      MECANGNG-NDLAETFCTQRAGPAPDPLNWNAAAESLKGSHLDEVKRMVDEFRRPLVRLG   59
PAL2      MENGHDEC-VKVSE-FCLK-----SDPLNWGVAAESLMGSHLDEVKRMVAEFRKPVVKFG   53
PAL3      MEHVHENGNVHHSNRFCVQ-----DDPLNWNAAAESLKGSHLDEVKKMVREYRKPLVKLG   55
          **  : :   . ::  ** :      *****..***** ********:** *:*:*:*::*

PAL1      GETLTIAQVAAVAASSDAA-VKVELSEGARAGVKASSDWVMESMRKGTDSYGITTGFGAT  118
PAL2      GESLTVAQVAAIAAKGDQG-VKVELAEDARAGVKASSDWVMESMNKGTDSYGVTTGFGAT  112
PAL3      GETLTVAQVAAVAAVHAGGGTVVELCESAKAGVKASSEWVVESMNNGTDSYGVTTGFGAT  115
          **:**:*****:**    . .  ***.*.*:*******:**:***.:******:*******

PAL1      SHRRTKQGGALQEELIRFLNAGIFGNGTETCHTLPHSATRASMLVRINTLLQGYSGIRFE  178
PAL2      SHRRTNQGGALQKELIRFLNAGIFGNGTETCHTLPHSATRAAMLVRINTLLQGYSGIRFE  172
PAL3      SHRRTKQGTALQTELIRFLNAGIFGNGTESGHTLPHSATRAAMLVRINTLLQGYSGIRFE  175
          *****:** *** ****************: **********:******************

PAL1      ILEAITKLLNNNITPCLPLRGTITASGDLVPLSYIVGLLTGRPNSKAVGPDGKFVNATEA  238
PAL2      ILEAITTFLNHNITPCLPLRGTITASGDLVPLSYIAGLLTGRPNSKAVGPNGEAFNAEEA  232
PAL3      ILEALTKLLNHNITPCLPLRGTITASGDLVPLSYIAGLLTGRPNSRCVGPNGRSLDATEA  235
          ****:*.:**:************************.*********:.***:*. .:* **

PAL1      FSLAGIDTGFFELQAKEGLALVNGTAVGSALASMVLFEANILAVLAEVLSGIFAEVMHGK  298
PAL2      FRLAGLSGGFFELQPKEGLALVNGTAVGSGLASIVLFEANVLAVLSVVLSAIFAEVMNGK  292
PAL3      FQIAGMNSGFFELQPKEGLALVNGTAVGSGLASMVLFEANLLVILSEVFSAIFAEVMHGK  295
          * :**:. ******.**************.***:******:*.:*: *:*.******:**

PAL1      PEFTDHLTHKLKHHPGQIEAAAIMEHILDGSSFVKEAQRVHEFDPLQKPKQDRYALRTSP  358
PAL2      PEFTDHLTHKLKHHPGQIEAAAIMEHILDGSSYVKAAQKLHELDPLQKPKQDRYALRTSP  352
PAL3      PEFIDHLMHKLKHHPGQIEAAAIMEHILDGSSYVKAAKALHETDPLQKPKQDRYALRTSP  355
          *** *** ************************:** *: :** *****************

PAL1      QWLGPLIEVIRASTKSIEREINSVNDNPLIDVSRNKALHGGNFQGTPIGVSMDNTRLAIA  418
PAL2      QWLGPQIEVIRAATKMIEREINSVNDNPLIDVSRNKALHGGNFQGTPIGVSMDNARLAIA  412
PAL3      QWLGPLIEVIRSSTKSIEREINSVNDNPLIDVSRNKALHGGNFQGTPIGVSMDNTRLAIA  415
          ***** *****::** **************************************:*****

PAL1      SIGKLMFAQFSELVNDFYNNGLPSNLSGGRNPSLDYGFKGAEIAMAAYCSELQFLANPVT  478
PAL2      SIGKLMFAQFSELVNDYYNNGLPSNLSGGRNPSLDYGFKGAEIAMAAYCSELQYLGNPVT  472
PAL3      SIGKLMFAQFSELVNDFYNNGLPSNLSGGRNPSLDYGFKGAEIAMAAYCSELQFLANPVT  475
          ****************:************************************:*.****

PAL1      NHVQSAEQHNQDVNSLGLISSRKTAEAVDILKLMSSTYLVALCQAIDLRHLEENLKASVK  538
PAL2      NHVQSAEQHNQDVNSLGLISSRKTAEAIDILKLMSSTYLVALCQAIDLRVLEENLKNAVK  532
PAL3      NHVQSAEQHNQDVNSLGLISSRKAAEAVDILKLMSSTYLVALCQAIDLRHLEENLKNAVK  535
          ***********************:***:********************* ****** :**

PAL1      NTVSLVAKKVLTMGYNGELHPSRFCEKDLLKVVDREHVFAYIDDPCSGTYPLMQKLRQVL  598
PAL2      NIVSQVAKRTLTMGANGELHPSRFCEKDLLRVVDREYAFAYVDDPCSATYPLMQKLRQVL  592
PAL3      STVHQVAKKVLTTGINGELHPSRFCEKDLLKVVEREYVFTYIDDPCSATYPLMQKLRQVL  595
          . *  ***:.** * ***************:**:**:.*:*:*****.************

PAL1      VEHSLANGDKEKDATTSIFQKIGAFEDELKALLPKEVESARCELENGKPGIANRIKDCRS  658
PAL2      VDHALKNGDQEKNVNTSIFQKIAAFEDELKAVLPKEVESARSAVESGNPAIPNRIRECRS  652
PAL3      VDHALIN-NEDLNPNTSIFLKVGAFEEELKTLLPIEVESARNACESGNPAVPNRIKKCRS  654
          *:*:* *  ::: : .**** *:.***:***::** ******   *.*:*.:.***:.***

PAL1      YSLYKFVRGELGTNFLTGEKVRSPGEEFDKVFTAICEGKLIDPLLDCLKEWNGAPRPIC  717
PAL2      YPLYKFVREVLGTELLSGEKAQSPGEVFDQVFTAMSKGQIVDPLLECLQEWNGAPLPIC  711
PAL3      YPLYKFVREDLATGFLTGEKAKSPGEEFDKVFSAICDGKMVDPLLECLKDWNGAPLPLC  713
          *.******  *.* :*:***.:**** **:**:*:..*:::****:**::***** *:*
```

Supplementary Fig. S1 Comparison of the amino-acid sequences encoded by the three *C. canephora* phenylalanine ammonia-lyases (*PALs*) genes. Multiple-sequence alignment was performed with ClustalW.

Identical amino acids in all three sequences are indicated by a star. The PAL active site has been highlighted in red, except for the serine residue, which is colored in blue. The Arg residue associated with the intron sequence is highlighted in pink
