# Supplementary material for: Characterization, high-resolution mapping and differential expression of three homologous PAL genes in Coffea canephora Pierre (Rubiaceae)
Source: Planta. 2012 Feb 21;236(1):313–26. doi: 10.1007/s00425-012-1613-2 (PMC3382651; doi:10.1007/s00425-012-1613-2)
Supplement: Supplementary file 2 — Supplementary material 2 (PDF 269 kb) [file 425_2012_1613_MOESM2_ESM.pdf]

Characterization, high-resolution mapping and differential expression of three homologous *PAL* genes in *Coffea canephora* Pierre (Rubiaceae).

Maud Lepelley[1,*], Venkataramaiah Mahesh[2,3], James McCarthy[1], Michel Rigoreau[1], Dominique Crouzillat[1], Nathalie Chabrillange[2], Alexandre de Kochko[2], Claudine Campa[2]

[1] Nestlé R&D Center, 101 Av. Gustave Eiffel, Notre Dame D'Oé, BP 49716, 37097 Tours, France

[2] IRD, UMR DIADE (IRD/UM2), BP 64501, 34394 Montpellier, France

[3] Avesthagen Limited, International Technology Park, Bangalore 560066, India

* Corresponding author E-mail Address: maud.lepelley@rdto.nestle.com

Supplementary Table S1 Primers and probes used in Taqman® real-time quantitative RT-PCR assay. Primers and probes were designed using the Primer Express software (Applied Biosystems). All MGB Probes were labelled at the 5' end with the fluorescent reporter dye 6-carboxyfluorescein (FAM) and at the 3' end with the quencher dye 6-carboxy-tetramethyl-rhodamine (TAMRA), except rpl39 probe which was labelled at the 5' end with the fluorescent reporter dye VIC and at the 3'end with quencher TAMRA. All sequences are given 5'-3'

Genes	Primers and Probes names	Primers and Probes sequences
rpl39	rpl39-F	GAACAGGCCCATCCCTTATTG
	rpl39-R	CGGCGCTTGGCATTGTA
	**rpl39-MGB**	**ATGCGCACTGACAACA**
PAL1	PAL1-F	GTTTGCCCTCTTTTTGGAATGTT
	PAL1-R	TATGGGACGAAAATACAAGGATCTTAA
	**PAL1-MGB**	**TCCAAGTTGTCACTAGCT**
PAL2	PAL2-F	GTCAACACCTCCATCTTCCAAAA
	PAL2-R	TGGTAGGACAGCCTTCAGTTCA
	**PAL2-MGB**	**ATTGCTGCATTTGAAG**
PAL3	PAL3-F	GCTCCGCTACCCTTGTGTTAA
	PAL3-R	CACCGAGTACAACAGCTAAAATCTG
	**PAL3-MGB**	**TCCACGGCACGTTGA**
HQT	HQT-F	TTGCCAAGTCCAGGCAAAG
	HQT-R	CATGTGATCGGCATCTAAGCA
	**HQT-MGB**	**CAGGACTTTATCGTTAGCTG**
