# Supplementary material for: Characterization, high-resolution mapping and differential expression of three homologous PAL genes in Coffea canephora Pierre (Rubiaceae)
Source: Planta. 2012 Feb 21;236(1):313–26. doi: 10.1007/s00425-012-1613-2 (PMC3382651; doi:10.1007/s00425-012-1613-2)
Supplement: Supplementary file 3 — Supplementary material 3 (PDF 300 kb) [file 425_2012_1613_MOESM3_ESM.pdf]

Characterization, high-resolution mapping and differential expression of three homologous *PAL* genes in *Coffea canephora* Pierre (Rubiaceae).

Maud Lepelley[1,*], Venkataramaiah Mahesh[2,3], James McCarthy[1], Michel Rigoreau[1], Dominique Crouzillat[1], Nathalie Chabrillange[2], Alexandre de Kochko[2], Claudine Campa[2]

[1] Nestlé R&D Center, 101 Av. Gustave Eiffel, Notre Dame D'Oé, BP 49716, 37097 Tours, France

[2] IRD, UMR DIADE (IRD/UM2), BP 64501, 34394 Montpellier, France

[3] Avesthagen Limited, International Technology Park, Bangalore 560066, India

* Corresponding author E-mail Address: maud.lepelley@rdto.nestle.com

Supplementary Table S2 List of all PAL protein sequences used to construct the phylogenetic tree presented in Fig. 2

Taxon	*Sequence name*	*Accession number*
A. Spermatophytes		
**1. Angiosperms**		
**Dicots**		
• **Rosids**		
Arabidopsis thaliana	AtPAL1 AEC09341	AEC09341
Arabidopsis thaliana	AtPAL2 AEE79055	AEE79055
Arabidopsis thaliana	AtPAL3 AED90715	AED90715
Arabidopsis thaliana	AtPAL4 AEE74893	AEE74893
Acacia mangium	AmPAL ABD42947	ABD42947
Brassica napus	BnPAL AAX22053	AAX22053
Brassica oleracea	BoPAL ADL09136	ADL09136
Cicer arietinum	CaPAL2 Q9SMK9	Q9SMK9
Cicer arietinum	CiaPAL CAB60719	CAB60719
Euphorbia pulcherima	EpPAL ACM44926	ACM44926
Garcinia mangostana	GamPAL ACM62741	ACM62741
Glycine max	GlmPAL1 CAA37129	CAA37129
Glycine max	GlmPAL2 ACS88364	ACS88364
Isatis tinctoria	ItPAL ABF50788	ABF50788
Jatropha curcas	JcPAL ABI33979	ABI33979
Litchi chinensis	LicPAL ACR15762	ACR15762
Lotus japonicus	LjPAL BAF36967	BAF36967
Manihot esculenta	MePAL1 AAK62030	AAK62030
Manihot esculenta	MePAL2 AAK60275	AAK60275
Medicago sativa	MsPAL CAA41169	CAA41169
Morus alba	MaPAL ADI40166	ADI40166
Pyrus communis	PcPAL ABB70117	ABB70117
Prunus avium	PraPAL AAC78457	AAC78457
Pisum sativum	PsPAL BAA00885	BAA00885
Pisum sativum	PsPAL2 Q04593	Q04593
Populus tremuloides	PtPAL AAN52279	AAN52279
Populus trichocarpa	PtrPAL1 ACC63888	ACC63888
Populus trichocarpa	PtrPAL2 ACC63890	ACC63890
Populus trichocarpa	PtrPAL3 ACC63887	ACC63887

Populus trichocarpa	PtrPAL4 ACC63891	ACC63891
Populus trichocarpa	PtrPAL5 ACC63889	ACC63889
Phaseolus vulgaris	PvPAL2 P19142	P19142
Phaseolus vulgaris	PvPAL3 P19143	P19143
Quercus suber	QsPAL AAR31107	AAR31107
Ricinus communis	RcPAL EEF30696	EEF30696
Rubus idaeus	RiPAL1 AAF40223	AAF40223
Rubus idaeus	RiPAL2 AAF40224	AAF40224
Robinia pseudoacacia	RpPAL ACF94716	ACF94716
Stylosanthes humilis	ShPAL AAA99500	AAA99500
Trifolium pratense	TpPAL AAZ29732	AAZ29732
Vitis vinifera	VvPAL ABM67591	ABM6759
• **Asterids**		
Ageratina	APAL1 ACT53398	ACT53398
Ageratina	APAL2 ACT53399	ACT53399
Beta vulgaris	BvPAL CAH17686	CAH17686
Camellia oleifera	CoPAL ACT21093	ACT21093
Camellia sinensis	CsPAL BAA05643	BAA05643
Capsicum annuum	CaaPAL ACF17667	ACF17667
Catharanthus roseus	CrPAL BAA95629	BAA95629
Cistanche deserticola	CdPAL ADD12041	ADD12041
Coffea canephora	CcPAL1	AAN32866
Coffea canephora	CcPAL2	AEO94540
Coffea canephora	CcPAL3	AEO94541
Cynara cardunculus	CycPAL3 CAL91169	CAL91169
Daucus carota	DcPAL D85850	D85850
Daucus carota	DcPAL2 BAG31930	BAG31930
Digitalis lanata	DlPAL CAA05251	CAA05251
Fagopyrum esculentum	FePAL ADC34597	ADC34597
Fagopyrum esculentum	FePAL2 ADT63058	ADT63058
Ipomea batatas	IbPAL1 AAA33389	AAA33389
Ipomea nil	InPAL AAG49585	AAG49585
Lactuca sativa	LsPAL AAL55242	AAL55242
Lactuca sativa	LsPAL2 AAO13347	AAO13347
Melissa officinalis	MoPAL CBJ23826	CBJ23826
Nicotiana attenuata	NaPAL1 ABG75910	ABG75910
Nicotiana attenuata	NaPAL2 ABG75911	ABG75911
Nicotiana tabacum	NtPAL1 P25872	P25872
Nicotiana tabacum	NtPAL2 P35513	P35513
Petunia hybrida	PhPAL1 AAV98199	AAV98199
Petrosilenum crispum	PecPAL1 P24481	P24481
Petrosilenum crispum	PecPAL3 CAA57057	CAA57057
Rehmannia glutinosa	RgPAL AAK84225	AAK84225
Rudbeckia hirta	RhPAL1 ABN79671	ABN79671
Scutellaria baicalensis	SbPAL ADN32766	ADN32766
Scutellaria baicalensis	SbPAL1 ADN32767	ADN32767
Scutellaria baicalensis	SbPAL2 ADN32768	ADN32768
Salvia miltiorrhiza	SmPAL ABD73282	ABD73282
Solanum tuberosum	StPAL1 P31425	P31425
Solanum lycopersicum	SlPAL1 P35511	P35511
Solanum lycopersicum	SlPAL5 P26600	P26600
Stellaria longipes	SlPAL AAR19393	AAR19393
Monocots		
Allium sepa	AsPAL AAS48415	AAS48415
Bambusa oldhamii	BoPAL1 ACX31738	ACX31738
Bromheadia finlaysoniana	BfPAL CAA68256	CAA68256
Lycoris radiata	LrPAL ACM61988	ACM61988
Musa acuminata	MuaPAL ACG56647	ACG56647
Musa balbisiana	MubPAL BAG70982	BAG70982
Oryza sativa	OsPAL2 A2X7F7	A2X7F7

Phyllostachys edulis	PePAL ABP96954	ABP96954
Triticum aestivum	TaPAL CAA68036	CAA68036
Zea mays	ZmPAL AAL40137	AAL40137
Paleodicots		
Persea americana	PeaPAL AAA51873	AAA51873
**2. Gymnosperms**		
Gingko biloba	GbPAL ABU49842	ABU49842
Ephedra sinica	EsPAL BAG74770	BAG74770
Pinus taeda	PtaPAL AAA84889	AAA84889
B. Tracheophytes		
Equisetum arvense	AePAL AAW80639	AAW80639
Blechnum spicant	BsPAL AAW80643	AAW80643
Isoetes lacustris	IlPAL AAW80637	AAW80637
Psilotum nudum	PnPAL AAW80640	AAW80640
Physcomitrella patens	PpPAL EDQ49523	EDQ49523
Selaginella kraussiana	SkPAL AAW80638	AAW80638
**Outgoup**		
Rhodotorula glutinis	RgPAL ABB04148	ABB04148
